# Influence of COD in Toxic Industrial Wastewater from a Chemical Concern on Nitrification Efficiency

**DOI:** 10.3390/ijerph192114124

**Published:** 2022-10-29

**Authors:** Iwona B. Paśmionka, Piotr Herbut, Grzegorz Kaczor, Krzysztof Chmielowski, Janina Gospodarek, Elżbieta Boligłowa, Marta Bik-Małodzińska, Frederico Márcio C. Vieira

**Affiliations:** 1 Department of Microbiology and Biomonitoring, Faculty of Agriculture and Economics, University of Agriculture in Krakow, 31-120 Krakow, Poland; 2 Department of Rural Building, Faculty of Environmental Engineering and Land Surveying, University of Agriculture in Krakow, 31-120 Krakow, Poland; 3 Biometeorology Study Group (GEBIOMET), Universida de Tecnológica Federal do Paraná (UTFPR), Estrada para Boa Esperança, km 04, Comunidade São Cristóvão, Dois Vizinhos 85660-000, Brazil; 4 Department of Sanitary Engineering and Water Management, Faculty of Environmental Engineering and Land Surveying, University of Agriculture in Krakow, 31-120 Krakow, Poland; 5 Institute of Soil Science, Engineering and Environmental Management, University of Life Sciences in Lublin, 20-069 Lublin, Poland

**Keywords:** chemical wastewater, activated sludge nitrifiers, inhibition of nitrification

## Abstract

COD is an arbitrary indicator of the content of organic and inorganic compounds in wastewater. The aim of this research was to determine the effect of COD of industrial wastewater on the nitrification process. This research covered wastewater from acrylonitrile and styrene–butadiene rubbers, emulsifiers, polyvinyl acetate, styrene, solvents (butyl acetate, ethyl acetate) and owipian^®^ (self-extinguishing polystyrene intended for expansion) production. The volume of the analyzed wastewater reflected the active sludge load in the real biological treatment system. This research was carried out by the method of short-term tests. The nitrification process was inhibited to the greatest extent by wastewater from the production of acrylonitrile (approx. 51%) and styrene–butadiene (approx. 60%) rubbers. In these wastewaters, nitrification inhibition occurred due to the high COD load and the presence of inhibitors. Four-fold dilution of the samples resulted in a two-fold reduction in the inhibition of nitrification. On the other hand, in the wastewater from the production of emulsifiers and polyvinyl acetate, a two-fold reduction in COD (to the values of 226.4 mgO_2_·dm^−3^ and 329.8 mgO_2_·dm^−3^, respectively) resulted in a significant decrease in nitrification inhibition. Wastewater from the production of styrene, solvents (butyl acetate, ethyl acetate) and owipian^®^ inhibited nitrification under the influence of strong inhibitors. Lowering the COD value of these wastewaters did not significantly reduce the inhibition of nitrification.

## 1. Introduction

Municipal and industrial wastewater is a carrier of many dangerous organisms and substances, which include parasites and pathogenic organisms [1,2,3], toxic substances and compounds disturbing the natural biological balance in treated sewage receivers by increasing the saprobicity and eutrophication of these waters [4,5,6]. Such wastewater can be neutralized by mechanical–biological or mechanical–biological–chemical methods of treatment with the use of activated sludge. Effective detection of any disruptions and instabilities in the processes taking place during wastewater treatment enables quick and effective actions to be taken to eliminate them, thus ensuring safety for the environment and human health [7,8,9,10].

The effectiveness of wastewater treatment by the activated sludge method depends on the appropriate balance between the number of nitrifying and heterotrophic bacteria, among other factors [11]. The dominant group of microorganisms in the activated sludge is determined by the quality and quantity of substrates contained in raw sewage and the availability of nutrients [12]. Nitrification is the most sensitive part of biological nitrogen removal from wastewater, with autotrophic nitrifying biomass being about 10 times more sensitive to inhibitory factors than its oxygen heterotrophic counterpart [13]. In the nitrification process, chemolithoautotrophic nitrosobacteria of the genera *Nitrosomonas*, *Nitrosovibrio*, *Nitrosococcus*, *Nitrosospira* and *Nitrosolobus* transform ammonium nitrogen to nitrite [14,15]. Then, nitrites are oxidized to nitrates with the participation of nitrobacteria of the genera *Nitrobacter*, *Nitrospira*, *Nitrococcus* and *Nitrospina* [16]. The critical nitrogen removal step is initiated by the conversion of ammonia to nitrate by the nitrifying microorganisms. Nitrification is considered to be a key step in biological wastewater treatment as it converts the toxic ammonia into nitrates, which can be further converted by denitrification into environmentally harmless gaseous forms of nitrogen. The nitrification process is sensitive to the presence of toxic substances in the wastewater. This is of particular importance in the case of industrial wastewater. These wastewaters, depending on the type of industry, are characterized by a different quantitative, and especially qualitative, composition. Many chemical compounds in this wastewater can inhibit the nitrification process. These compounds include, among others: butadiene, styrene, alkylbenzenesulfonic acid, acetic acid, butylcatechol, tricresol, acetonitrile, alkyldimethylbenzylammonium chloride, alkyldimethylbenzylammonium bromide and many other substances, which have been widely documented in the literature. Many of these compounds are quaternary ammonium compounds that found widespread use as disinfectants during COVID-19. They induce a reduction in the intensity of nitrification or even put a complete stop to this process [17,18].

Inhibition of nitrification in industrial wastewater can be attributed to the presence of compounds with hydrophobic properties, a high molecular weight and aromatic structures [19]. According to Pagg et al. [20], inhibition of nitrification depends on the level of biodegradability of the potential inhibitory compound and is less severe in the case of biodegradable substances. The biodegradability of wastewater may be indicated by the ratio of COD to BOD_5_ [21]. In order to consider raw sewage as susceptible to biodegradation, the value of the COD/BOD_5_ relationship, according to Siwiec et al. [22], Samudro et al. [23] and Bader et al. [24], should be lower than 2.2 or even 2.0. On the other hand, the ratio of the concentration of easily digestible organic compounds to the concentration of total nitrogen and total phosphorus is decisive for the possible effectiveness of nutrient removal from municipal wastewater [25].

The growth rate of nitrifying bacteria is influenced by temperature, substrate concentration, oxygen content, pH and the presence of toxic inhibitory compounds, i.e., inhibitors. In a study conducted by Bawiec [26], free ammonia was a factor that inhibited the metabolism of bacteria of the genus *Nitrobacter*. The amount of free ammonia in sewage depends on the concentration of ammonium ions, temperature and pH of the sewage [5]. The production of excessive ammonia nitrogen emissions is a common problem in many types of industrial wastewater [19].

Heavy metals are considered to be very strong inhibitors of nitrification. The influence of cadmium, copper and mercury on *Nitrosomonas europaea* in quasi-stationary batch reactors is well-known [27]. Ouyang et al. [28] demonstrated in their research that the increased concentration of Cu in wastewater inhibited the oxidation of ammonium nitrogen rather than nitrite.

Inhibition of nitrification in mechanical–biological municipal and industrial wastewater treatment plants may cause problems resulting from the non-conversion of the ammonium form of nitrogen and thus a high concentration of this ion in the wastewater. Inhibition problems may sometimes occur periodically, which makes it very difficult to find the source of substances inhibiting the nitrification process [29]. The factor influencing the inhibition of nitrification may also be excessive salinity of the wastewater. It causes plasmolysis or a reduction in the activity of organisms. In the presence of salt, there is a lower efficiency of biological nitrogen removal processes, including nitrification, and especially denitrification. According to Dincer and Karga [30], a salt concentration above 2% causes a significant reduction in the efficiency of both nitrification and denitrification, and the authors confirmed in their research that denitrification was more sensitive to salinity than nitrification.

In the case of industrial wastewater from coking plants, although the activated sludge process has been adapted to their treatment, nitrification is often disturbed by high concentrations of chemical compounds, expressed in the COD value. Kim et al. [31] investigated the inhibitory effect of ammonia, thiocyanate, iron cyanide, phenol and *p*-cresol on nitrification in the activated sludge treatment technology. The authors showed that ammonia in a concentration below 350 mg·dm^−3^ did not limit the substrate for nitrifying bacteria. On the other hand, a concentration of thiocyanate above 200 mg·dm^−3^ significantly inhibited nitrification, but iron cyanide with a concentration below 100 mg·dm^−3^ did not. Nitrification was also inhibited by phenol with a concentration above 200 mg·dm^−3^ and *p*-cresol with a concentration above 100 mg·dm^−3^. In the studies on the treatment of wastewater from chemical production, carried out by Paśmionka and Gospodarek [17], it was found that the nitrification process was inhibited to the greatest extent (72%) by wastewater from styrene–butadiene rubber production. On the other hand, wastewater from the production of methacrylate (polymethyl methacrylate) had the lowest degree of inhibition (16%). The investigated sewage also had a toxic effect on the entire biocenosis and adversely affected the structure of activated sludge flocs. According to Ge et al. [32], measuring the effect of potential inhibitors on the nitrification rate is important for maintaining the appropriate efficiency and effectiveness of mechanical–biological wastewater treatment plants. Juliastuti et al. [33], on the basis of research carried out on industrial wastewater containing selected organic compounds, found that the degree of nitrification inhibition was reduced by the compounds in the following order: chlorobenzene > trichlorethylene > phenol > ethylbenzene. Chlorobenzene, even at the level of 0.25 mg·dm^−3^, reduces the autotrophic biomass. The nitrification process is completely inhibited by chlorobenzene at the concentration of 0.75 mg·dm^−3^. Trichlorethylene (TCE) has a less inhibitory effect on the nitrification process. The increase to 50% of inhibition is observed only at the concentration of 0.75 mg·dm^−3^ TCE. The nitrification process is completely inhibited at the concentration of 1 mg·dm^−3^ TCE. Phenol inhibits nitrification by 50% at 3 mg·dm^−3^. The inhibitory effect of phenol is almost constant in the range of 4–10 mg·dm^−3^, and the complete inhibition of this process is achieved at 50 mg·dm^−3^. The inhibitory effect of ethylbenzene is 50% at 8 mg dm^−3^, and the autotrophic biomass is completely inactive at 50 mg·dm^−3^. In a study by Park et al. [34], the effect of phenol on nitrification was assessed. After 400 mg·dm^−3^ of phenol was added to the sewage, the NO_3_-N concentration in the sewage outflow decreased from 69.24 and 51.24 mg·dm^−3^ to 1.89 and 1.51 mg·dm^−3^, respectively, within 14 days. After lowering the phenol concentration to 60 mg·dm^−3^, the efficiency of nitrification gradually increased.

The aim of this research was to determine the effect of the concentration of organic and inorganic pollutants (expressed in COD value) in industrial wastewater on the degree of inhibition of nitrification. The study covered wastewater from acrylonitrile and styrene–butadiene rubbers, emulsifiers, polyvinyl acetate, styrene, solvents (butyl acetate, ethyl acetate) and owipian^®^ (self-extinguishing polystyrene intended for expansion) production.

## 2. Materials and Methods

### 2.1. Description of the Research Object

The research was conducted in the Municipal and Industrial Sewage Treatment Plant, located in the city of Oświęcim in the Monowice district (50°02′17.1″ N 19°19′13.8″ E) in Lesser Poland. The sewage treatment plant is supplied with municipal sewage from the city and commune of Oświęcim as well as industrial sewage from the Synthos chemical plant.

The planned capacity of the sewage treatment plant is 53,400 m^3^·d^−1^, with industrial sewage amounting to 26,400 m^3^·d^−1^, while municipal sewage amounts to 27,000 m^3^·d^−1^. The facility’s technology is based on the processes of mechanical, chemical and biological treatment of industrial and municipal wastewater, using the activated sludge method. Municipal and industrial wastewater is pretreated in separate technological lines consisting of grates, sand traps, degreasers and primary sedimentation tanks. Its purpose is to separate floating and dragged pollutants, sand, fats, organic suspension from sewage and to correct the pH to a value that allows for their further biological treatment. Industrial wastewater is additionally neutralized and coagulated in a mixer system. Pretreated wastewater streams are mixed and directed to biological treatment. The biological treatment system uses an anaerobic chamber, four aeration chambers, three secondary radial settling tanks, a blower station and an activated sludge-pumping station. Wastewater treatment takes place in two stages:

- The first stage (anaerobic) is carried out in an anaerobic chamber to which sewage, free of dissolved oxygen, is supplied. This stage guarantees the occurrence of favorable conditions conducive to the development of anaerobic organisms responsible for the reduction processes.
- The second stage (aerobic) takes place in aeration chambers equipped with agitators and an installation for fine-bubble aeration with compressed air. The concentration of dissolved oxygen in the aerobic chambers ranges from 1.5 to 2.5 mg·dm^−3^, which favors the development of organisms responsible for the aerobic decomposition of pollutants.

Treated sewage is separated from activated sludge in secondary radial settling tanks. After the sedimentation process, the activated sludge is collected in the central hopper of the secondary radial settling tank, from where it then flows into the chamber, from which it is returned to the process as recirculated sludge or is pumped out as excess sludge. The treated wastewater is collected through the overflow troughs on the outskirts of the settling tanks, and from there it is discharged to the receiver, which is the Macocha stream, a tributary of the Vistula.

Active sludge cultivated in the presence of pretreated industrial sewage from the Synthos chemical plant, mixed with municipal sewage, was used in the conducted research. Table 1 presents the average values of technological parameters of the activated sludge used during the research.

### 2.2. Research Description

The tests were carried out in static conditions, during a four-hour aeration process of properly prepared activated sludge and tested sewage. Wastewater from plants producing individual organic compounds was analyzed. Before starting the tests, the activated sludge (in the amount of 1 dm^3^) was centrifuged and then washed with an aqueous solution of sodium bicarbonate and ammonium sulfate. The thus-prepared pellet was diluted with distilled water in the ratio 1:10 and centrifuged again. The activated sludge was suspended in the appropriate volume of tap water in order to obtain the concentration required for the tests. Then, the medium was prepared by dissolving 5.04 g of NaHCO_3_ and 2.64 g of (NH_4_)_2_SO_4_ in 1 dm^3^ of distilled water. Further details are provided below:

(a) Control flask contents:
  - Activated sludge: 125 cm^3^;
  - Medium: 25 cm^3^;
  - Distilled water: 100 cm^3^.
(b) Comparative flask contents:
  - Activated sludge: 125 cm^3^;
  - Medium: 25 cm^3^;
  - Reference inhibitor, allylthiourea (ATU): 2.5 cm^3^;
  - Distilled water: 97.5 cm^3^.
(c) Test flask contents:
  - Activated sludge: 125 cm^3^;
  - Medium: 25 cm^3^;
  - Tested sewage in the amount of 25 cm^3^, 50 cm^3^, 75 cm^3^ and 100 cm^3^;
  - Distilled water in the amount of 75 cm^3^, 50 cm^3^, 25 cm^3^ and 0 cm^3^ (according to the amount of sewage tested).

The volume of the mixture in each flask was 250 cm^3^. The systems prepared in this way were aerated for 4 h with moist, compressed air using VEB ELMET TYP Fp 09 pumps. The concentration of dissolved oxygen in the systems was about 2 mg·dm^−3^. Immediately after 4 h of aeration, a sample was taken from each flask, and the concentration of NH_4_-N, NO_2_-N and NO_3_-N was determined after filtering. The test results are expressed as the percentage of nitrification inhibition (IN), calculated in relation to the control flask and to the flask with the comparative inhibitor, according to Formula (1):

(1) $$\%IN=\frac{C_{C}-C_{T}}{C_{C}-C_{B}}\cdot 100$$

in which:

$C_{C}$—concentration of oxidized forms of nitrogen (NO_2_-N + NO_3_-N) in the control flask after 4 h of aeration [mg·dm^−3^];
$C_{T}$—concentration of oxidized forms of nitrogen (NO_2_-N + NO_3_-N) in the flask with the tested sewage, after 4 h of aeration [mg·dm^−3^];
$C_{B}$—concentration of oxidized forms of nitrogen (NO_2_-N + NO_3_-N) in a flask with a comparative inhibitor (ATU) after 4 h of aeration [mg·dm^−3^].

Ahlstrom Munktell 389 filters were used to filter the samples.

The study used wastewater inhibiting the nitrification process to a degree higher than 30.0%, coming from the production of styrene–butadiene and acrylonitrile rubbers, styrene, solvents (butyl acetate, ethyl acetate), emulsifiers, polyvinyl acetate and owipian^®^. Wastewater in the amount of 25 cm^3^, 50 cm^3^, 75 cm^3^ and 100 cm^3^ was used for the tests, with a variable COD value.

### 2.3. Determination of NH_4_-N, NO_2_-N and NO_3_-N Concentrations

The concentrations of NH_4_-N, NO_2_-N and NO_3_-N were determined by the colorimetric method using the CADAS 30S biochemical spectrophotometer (Dr Lange, The Nederland) and appropriate cuvette tests. Measurements were made in triplicate.

### 2.4. Calculation of the Nitrification Activity of the Activated Sludge and the Specific Rate of Nitrification

The nitrifying activity of the activated sludge was calculated based on the differences in the concentration of oxidized forms of nitrogen (NO_3_-N and NO_2_-N) in the control flask and the flask with the comparative inhibitor, after 4 h of aeration, using Formula (2):

(2) $$R=\frac{C_{C}-C_{B}}{MLVSS\cdot 4}$$

in which:

R—nitrification activity of activated sludge [mg·g·h^−1^];
$C_{C}$—concentration of oxidized forms of nitrogen (NO_2_-N + NO_3_-N) in the control flask after 4 h of aeration [mg·dm^−3^];
$C_{B}$—concentration of oxidized forms of nitrogen (NO_2_-N + NO_3_-N) in a flask with a comparative inhibitor (ATU), after 4 h of aeration [mg·dm^−3^];
MLVSS—mixed liquor volatile suspended solids [mg·dm^−3^].

The specific rate of nitrification was determined on the basis of changes in NH_4_-N concentration in the control flask and the flask with the comparative inhibitor, after 4 h of aeration, according to Formula (3):

(3) $$S=\frac{C{\left(NH_{4}-N\right)}_{B}-C{\left(NH_{4}-N\right)}_{C}}{MLVSS\cdot 4}$$

in which:

S—specific rate of nitrification [mg·g·h^−1^];
$C{\left(NH_{4}-N\right)}_{B}$—ammonium nitrogen concentration in a flask with a comparative inhibitor (ATU) after 4 h of aeration [mg·dm−^3^];
$C{\left(NH_{4}-N\right)}_{C}$—ammonium nitrogen concentration in the control flask, after 4 h of aeration [mg·dm^−3^];
MLVSS—mixed liquor volatile suspended solids [mg·dm^−3^].

### 2.5. Verification of Measurement Data

Before the statistical analysis was performed, the measurement data were verified with regard to outliers. In order to apply appropriate parametric or nonparametric tests of outlier detection, at the beginning, the statistical distributions of individual observational data strings were assessed. The COD values, expressed in mg·dm^−3^, and the values of the nitrification inhibition parameter (IN), expressed in%, were analyzed. The normality of individual data distributions was tested with the Shapiro–Wilk test. In the null hypothesis H_0_ for this test, it was assumed that the given research sample comes from a normally distributed population. The significance level for this test was assumed to be α = 0.05. The results of the Shapiro–Wilk test for measurement data, including COD and IN values, are presented in Table 2.

Since for each data string, the assumed significance level α = 0.05 is lower than the calculated value of the test probability *p*-value, there is no reason to reject the null hypothesis that the distribution of individual data is normal. The normality of the distribution of measurement data allowed for the verification of outliers by applying the parametric Grubbs’ T-test. The calculations were performed with the statistical package Statistica v. 13.1 (StatSoft, Tulsa, OK, USA). The results of the Grubbs’ T-test are summarized in Table 3.

The data in Table 3 show that for each analyzed measurement data sequence, there are no grounds to reject the null hypothesis H_0_ stating that there are no outliers in a given data sequence.

### 2.6. Linear Regression Analysis

Statistical analysis of linear regression was used to determine the strength and direction of the impact of the COD value in industrial wastewater on the process of nitrification inhibition. The independent variable in this analysis was the value of COD in the wastewater, defined in mgO_2_ dm^−3^, while the dependent variable was the parameter IN, given as a percentage, calculated according to Formula (1). The relationship between the tested parameters in individual types of industrial wastewater is described by Equation (4):

IN = a·COD + b(4)

The parameters of the linear regression model were determined by the least-squares method. The significance of the relationship between the analyzed variables, the significance of the slope coefficient of the regression equation and the significance of the coefficient of determination R^2^ were verified by the Fisher–Snedecor F-test. Statistical calculations, along with the verification and estimation of the parameters of the regression equation, were performed with the Statistica v. 13.1 computer program (StatSoft, Tulsa, OK, USA).

## 3. Results

In studies conducted on wastewater from acrylonitrile and styrene–butadiene rubber production, the inhibition of nitrification occurred due to the concentration of pollutants (expressed in the COD value) contained in both wastewater samples and the presence of inhibitors. In the wastewater from the production of acrylonitrile rubbers, a two-fold reduction in the COD value resulted in a reduction in nitrification inhibition from 50.7% in the initial sample to 19.8%. As the wastewater was diluted further, the inhibitory effect was further reduced (Table 4).

In the wastewater from styrene–butadiene rubber production, a four-fold dilution of the initial sample with a COD of 627.2 mgO_2_·dm^−3^ caused only a two-fold reduction in nitrification inhibition (from 59.8% to 28.6%). Therefore, it can be concluded that reducing the concentration of pollutants in the tested sample to the COD value of 156.8 mgO_2_·dm^−3^ does not satisfactorily reduce the inhibition observed in the sample with the maximum concentration of pollutants (Table 5).

Tests conducted on wastewater from emulsifiers and polyvinyl acetate production have shown that in this case, the nitrification process was limited by a high COD load. However, even a two-fold dilution of this wastewater significantly reduced the degree of inhibition, and its further dilution caused its gradual decrease. In the case of wastewater from the production of emulsifiers, a two-fold reduction in COD (to the value of 226.4 mgO_2_·dm^−3^) compared to the initial sample resulted in a decrease in nitrification inhibition from 44.5% to 17.0% (Table 6).

In the wastewater from polyvinyl acetate production, a two-fold reduction in the COD concentration (to the value of 329.8 mgO_2_·dm^−3^) resulted in a decrease in nitrification inhibition from 38.9% to 16.1% (Table 7).

Tests conducted on wastewater from styrene, solvents (butyl acetate, ethyl acetate) and owipian^®^ production indicate that the inhibition of the nitrification process was due to the presence of a strong inhibitor or many inhibitors of this process in the tested wastewater. The percentage of nitrification inhibition did not decrease with dilution of this wastewater, but remained more or less constant (despite the continuous reduction in the COD value). In the case of sewage from styrene production, the inhibition of nitrification in the initial sample was slightly lower (45.1%, with a COD of 242.1 mgO_2_·dm^−3^), but after four-fold dilution, it remained at a relatively high level, 37.5%, with a COD of only 60.5 mgO_2_·dm^−3^ (Table 8).

The four-fold dilution of wastewater from solvent (butyl acetate, ethyl acetate) production, despite the decrease in COD from 491.6 mgO_2_·dm^−3^ to 122.9 mgO_2_·dm^−3^, did not remove the inhibition effect. The degree of inhibition of the nitrification process remained at the level of approx. 50.0%; thus, it was comparable to the initial sample (Table 9).

A similar result was obtained when testing industrial wastewater from owipian^®^ production. The reduction in the concentration of organic pollutants in the form of COD from 621.3 mgO_2_·dm^−3^ to 155.3 mgO_2_·dm^−3^ did not reduce the inhibition of nitrification, which still amounted to approx. 50.0% (Table 10).

**Figure 1:**
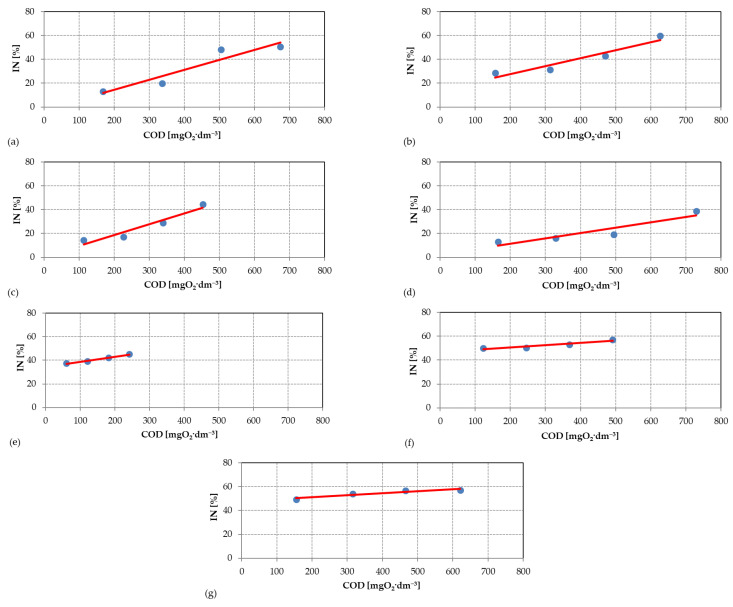
Scatterplots of measurement data from laboratory tests against established regression lines: (**a**) wastewater from acrylonitrile rubber production; (**b**) wastewater from styrene–butadiene rubber production; (**c**) wastewater from emulsifier production; (**d**) wastewater from polyvinyl acetate production; (**e**) wastewater from styrene production; (**f**) wastewater from solvent (butyl acetate, ethyl acetate) production; (**g**) wastewater from owipian^®^ production.

Regression analysis was used to test and statistically confirm the relationship between the COD values in individual industrial wastewaters and the IN parameter. Figure 1 shows the scatterplots of measurement data determined from laboratory tests against the regression lines calculated using the least-squares method.

The results of the calculations of the individual parameters of the regression line are summarized in Table 11. The *p*-value is marked in red, which indicates the statistical irrelevance of most often one of the coefficients of the equation for the significance level of 0.05. Although regression models describing the dependence of COD and IN in wastewater from acrylonitrile rubbers, polyvinyl acetate and owipian^®^ production are not statistically certain, high values of R and R^2^ confirm a strong correlation between the parameters studied. Additionally, it is worth noting that the greatest statistical uncertainty of the regression model was found to be 6.8% for industrial wastewater from the production of polyvinyl acetate, with the assumed uncertainty level of 5.0%. It follows that the regression line describes 93.2% of the measurement data. The deviation from the assumed materiality level is only 1.8%.

**Figure 2:**
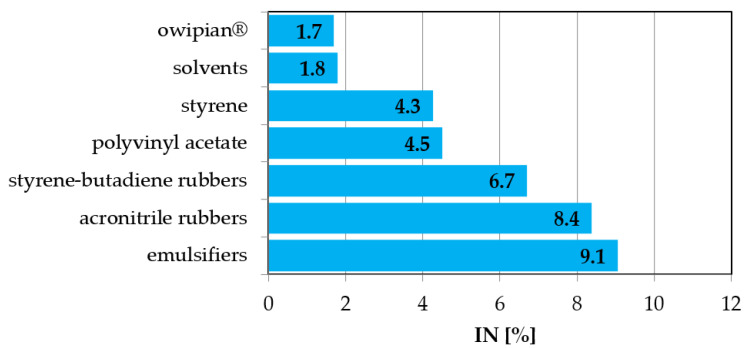
Percentage reduction in nitrification inhibition while reducing the COD value in industrial wastewater by 100 mgO_2_·dm^−3^.

Values of the slope of the regression line “a” in the second column of Table 11 indicate the strength of the influence of COD on the value of the IN parameter. This relationship is presented graphically in Figure 2. The individual bars of the graph show by what percent the inhibition of nitrification will decrease, expressed by the value of the IN parameter as a percentage, when the COD value in industrial wastewater drops by 100 mgO_2_·dm^−3^. The presented dependence shows that the pollutants expressed by the COD value most strongly inhibit the nitrification process during the production of emulsifiers and acrylonitrile rubbers, and inhibit it the least during the production of owipian^®^ and solvents.

**Figure 3:**
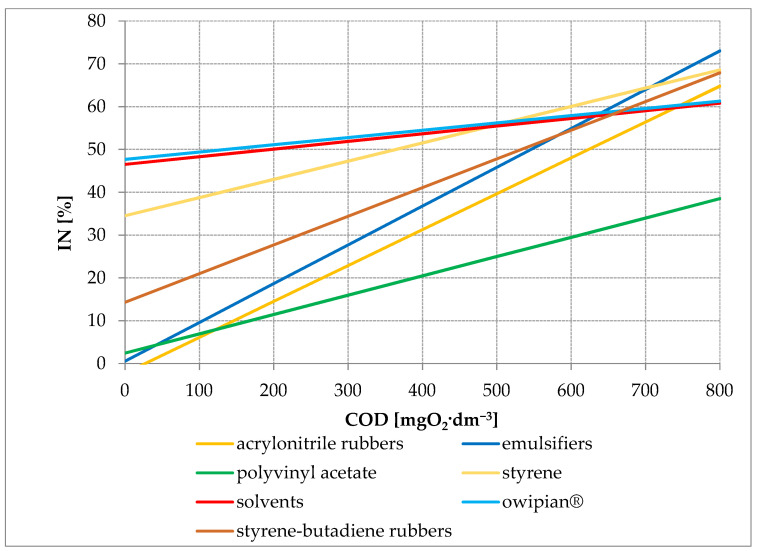
The course of the regression line showing the effect of the COD value in industrial wastewater on the nitrification inhibition expressed by the value of the IN parameter.

Figure 3 presents the course of the regression lines showing the effect of COD values in industrial wastewater on the inhibition of nitrification expressed by the value of the IN parameter. The presented dependencies indicate that during the production of acrylonitrile rubbers, polyvinyl acetate and emulsifiers, the inhibition of nitrification decreases proportionally with the decrease in the COD value in the wastewater. At very low COD values, the nitrification inhibition process stops. On the other hand, in the case of the production of solvents (butyl acetate, ethyl acetate), with a complete reduction in COD, the inhibition remains constant at 49.9%, and similarly, it remains at 49.4% in the case of the production of owipian^®^ and 37.5% in the production of styrene. It can be concluded from these values that the substances inhibiting the nitrification process are not directly related to the pollutants included in the COD group. These are probably the inhibitory substances found in the tested wastewater.

## 4. Discussion

The constantly developing chemical industry affects the degradation of the natural environment, the protection of which requires large financial outlays related to the necessity of neutralizing the resulting wastewater. Pretreated wastewater from production plants is usually directed to a mechanical and biological wastewater treatment plant [35]. Despite pretreatment, this sewage contains numerous organic and inorganic compounds, the concentration of which expresses the COD value. High COD values have a negative impact on purification processes, including nitrification, which was confirmed by our research. COD values exceeding 400 mgO_2_·dm^−3^ caused nitrification inhibition above 50%. COD values below 200 mgO_2_·dm^−3^ only slightly reduced the efficiency of this process, with the exception of sewage from the production of styrene, solvents (butyl acetate, ethyl acetate) and owipian^®^. In this case, it is likely the factors limiting nitrification were the inhibitors present in the tested chemical sewage. It is also possible that this effect was increased by numerous disinfecting compounds present in municipal wastewater, used during COVID-19 [36].

According to the literature data, the amount of nitrogen removed from the system in the form of biomass ranges from 20 to 30% [12]. These data, however, are not reflected in the biological system of the Municipal and Industrial Sewage Treatment Plant in Oświęcim, in which the amount of nitrogen removed from the system as a result of assimilation is only 12.4% [5]. It should be emphasized that the proportion of carbon to nitrogen incorporated in the biomass in this system is 31:1, which corresponds to the average values given in the available literature. This proves that carbon is not a limiting factor, as the minimum C:N demand ranges from 10:1 to 17:1 [12,21,25]. Limiting the sludge growth and the related decrease in the intensity of nitrification, as observed in our research, is most often the response of activated sludge to dynamic changes in the external environment, which are the cause of a long process of biochemical and physical adaptation in microorganisms, which was also observed by Rahimi et al. [37].

Significant factors limiting or even preventing the course of nitrification in the biological treatment system of the Municipal and Industrial Sewage Treatment Plant in Oświęcim are inhibitors flowing in with industrial sewage and high COD values. The list of organic and inorganic compounds having a negative effect on nitrifying bacteria is very long and includes a number of substances for which the degree of inhibition has been determined [6,13,17,19,20]. Most of these compounds were present in the sewage we analyzed. These substances are much more toxic to nitrifying bacteria than activated sludge heterotrophs. Most industrial wastewater contains various compounds, which, alone or in a mixture, have an inhibitory effect on nitrification [29].

As part of this study, the degree of nitrification inhibition under the influence of various concentrations of chemical pollutants (determined by the COD value) present in selected industrial wastewater was assessed. The research was carried out to determine the COD value in postproduction wastewater, which inhibited the nitrification process. The obtained results clearly show that the high concentration of organic pollutants in most of the tested wastewater was one of the main reasons for a significant reduction in nitrification. This is evidenced by the results obtained during the testing of wastewater from acrylonitrile and styrene–butadiene rubbers, styrene, solvents (butyl acetate, ethyl acetate) and owipian^®^ production. In the tested sewage, sometimes even a four-fold reduction in the concentration of organic pollutants, expressed in the form of COD, did not satisfactorily eliminate the inhibition process. Similar observations were made by Samudro et al. [23], who examined industrial wastewater from the production of urea, urea–formaldehyde adhesives, butanol, 2-ethylhexanol, maleic and phthalic anhydride and adhesive resins.

The conducted research showed that in the biological wastewater treatment system at the Municipal and Industrial Wastewater Treatment Plant in Oświęcim, the nitrification process is clearly disturbed by high COD values and the presence of inhibitory substances in the incoming wastewater.

The diversified production profile in the chemical industry hinders the proper operation of mechanical and biological wastewater treatment plants. In such plants, the amount and composition of sewage as well as the concentration of organic pollutants expressed in the COD value are subject to continuous fluctuations [38,39]. The biocenosis of activated sludge is sensitive to chemical substances [40]; therefore, treatment plants have difficulties in removing toxins and minimizing the concentration of organic pollutants in the incoming sewage [41,42]. This inhibits many processes, including nitrification, which was observed at the WWTP in Oświęcim.

## 5. Conclusions

Industrial wastewater contains numerous chemicals, including toxic ones, which significantly contribute to the inhibition of the nitrification process. In the Municipal and Industrial Wastewater Treatment Plant in Oświęcim, the most dangerous industrial wastewater comes from the production of acrylonitrile and styrene–butadiene rubbers, emulsifiers, polyvinyl acetate, styrene, solvents (butyl acetate, ethyl acetate) and owipian^®^. The main reason for the low efficiency of the nitrification process at the WWTP in Oświęcim is the concentration of organic pollutants present in the sewage and the presence of inhibitors. COD values exceeding 400 mgO_2_·dm^−3^ caused nitrification inhibition above 50%. COD values below 200 mgO_2_·dm^−3^ only slightly reduced the efficiency of this process, with the exception of sewage from the production of styrene, solvents (butyl acetate, ethyl acetate) and owipian^®^. Diluting industrial wastewater with municipal wastewater at a ratio of 1:3, or even 1:1, has a positive effect on the treatment processes, but still does not guarantee the elimination of nitrification inhibition. At present, industrial wastewater destined for biological treatment is mixed with municipal wastewater in a proportion of 2:1 or even 3:1, and it is not possible to dilute it further, due to the insufficient volume of available municipal wastewater. Only a clear advantage of typical domestic sewage in the system would allow for a partial reduction in nitrification inhibition. Nitrifying bacteria present in the activated sludge of the WWTP in Oświęcim are subject to the phenomenon of chronic stress. This is evidenced by the lower nitrification activity of activated sludge than in the literature, which in the period studied was on average about 1.0 mg·g·h^−1^ of oxidized nitrogen. It should be emphasized, however, that under favorable conditions, this activity is sufficient to maintain the parameters of the water-legal permit for the removal of ammoniacal nitrogen from wastewater.

## Figures and Tables

**Table 1:** Technological parameters of the activated sludge used for the research at the Municipal and Industrial Sewage Treatment Plant in Oświęcim.

Parameter	Unit	Value
temperature	°C	14.6
oxygenation	mgO_2_·dm^−3^	1.5–3
aeration time	h	4–5
total suspension of the activated sludge	mg·dm^−3^	3200–5300
total suspension in activated excess sludge	mg·dm^−3^	6000–8000
increase in excess activated sludge	m^3^·d^−1^	300–420
recirculation	% in relation to the incoming sewage	95–120
dry mass of activated sludge	%	1.69
age of the activated sludge	d	13–14
load BOD_5_	kgBOD_5_·kg_d.m._·d^−1^	0.13–0.28

**Table 2:** The results of the assessment of the normality of the distribution of data including COD and IN values (Shapiro–Wilk test).

Production Line	Shapiro–Wilk Normality Test Results for COD			Shapiro–Wilk Normality Test Results for IN		
	W	α	*p*-Value	W	α	*p*-Value
acrylonitrile rubbers	1.000	0.05	1.000	0.808	0.05	0.133
styrene–butadiene rubbers	1.000		1.000	0.987		0.785
emulsifiers	1.000		0.995	0.993		0.844
polyvinyl acetate	0.990		0.806	0.850		0.240
styrene	1.000		1.000	1.000		1.000
solvents (butyl acetate, ethyl acetate)	1.000		0.999	0.986		0.774
owipian^®^	1.000		0.630	0.861		0.269

W—the value of the Shapiro–Wilk test statistic. α—significance level, 0.05. *p*-value—the value of the calculated test probability.

**Table 3:** The results of the Grubbs’ T-test for the verification of outliers in the measurement series of COD and IN values.

Production Line	Grubbs’ T-Test Results for COD			Grubbs’ T-Test Results for IN		
	G	α	*p*-Value	G	α	*p*-Value
acrylonitrile rubbers	1.000	0.05	1.000	1.152	0.05	0.133
styrene–butadiene rubbers	1.000		1.000	1.059		0.785
emulsifiers	1.001		0.995	1.044		0.844
polyvinyl acetate	1.053		0.806	1.146		0.240
styrene	1.000		1.000	1.000		1.000
solvents (butyl acetate, ethyl acetate)	1.000		0.999	1.061		0.774
owipian^®^	1.006		0.980	1.143		0.269

G—the value of Grubbs’ T-test statistic. α—significance level, 0.05. *p*-value—the value of the calculated test probability.

**Table 4:** Influence of COD of industrial wastewater from acrylonitrile rubber production on the nitrification inhibition in activated sludge under laboratory conditions.

Samples	pH	COD (mgO_2_·dm^−3^)	Concentration NH_4_-N (mg·dm^−3^)		Concentration NO_3_-N (mg·dm^−3^)		% IN
			Before Aeration	After 4 h of Aeration	Before Aeration	After 4 h of Aeration	
control	7.6	-	56.00	23.00	0.00	15.60	-
ATU	7.6	-	56.00	49.00	0.00	0.00	-
sewage 25 cm^3^	7.2	168.4	56.00	23.00	0.00	13.54	13.2
sewage 50 cm^3^	7.2	336.7	56.00	29.00	0.00	12.51	19.8
sewage 75 cm^3^	7.2	505.1	56.00	31.40	0.00	8.07	48.3
sewage 100 cm^3^	7.2	673.4	56.00	29.20	0.00	7.69	50.7

MLVSS—4.700 g·dm^−3^, R—0.45, S—0.75.

**Table 5:** Influence of COD of industrial wastewater from styrene–butadiene rubber production on the nitrification inhibition in activated sludge under laboratory conditions.

Samples	pH	COD (mgO_2_·dm^−3^)	Concentration NH_4_-N (mg·dm^−3^)		Concentration NO_3_-N (mg·dm^−3^)		% IN
			Before Aeration	After 4 h of Aeration	Before Aeration	After 4 h of Aeration	
control	7.6	-	56.00	22.60	0.00	14.08	-
ATU	7.6	-	56.00	37.40	0.00	0.00	-
sewage 25 cm^3^	7.9	156.8	56.00	26.10	0.00	10.05	28.6
sewage 50 cm^3^	7.9	313.6	56.00	27.20	0.00	9.69	31.2
sewage 75 cm^3^	7.9	470.4	56.00	29.90	0.00	8.07	42.7
sewage 100 cm^3^	7.9	627.2	56.00	33.00	0.00	5.66	59.8

MLVSS—2.910 g·dm^−3^, R—1.21, S—1.27.

**Table 6:** Influence of COD of industrial wastewater from emulsifiers production on the nitrification inhibition in activated sludge under laboratory conditions.

Samples	pH	COD (mgO_2_·dm^−3^)	Concentration NH_4_-N (mg·dm^−3^)		Concentration NO_3_-N (mg·dm^−3^)		% IN
			Before Aeration	After 4 h of Aeration	Before Aeration	After 4 h of Aeration	
control	7.6	-	56.00	22.60	0.00	14.08	-
ATU	7.6	-	56.00	37.40	0.00	0.00	-
sewage 25 cm^3^	7.5	113.2	56.00	29.10	0.00	12.06	14.3
sewage 50 cm^3^	7.5	226.4	56.00	30.70	0.00	11.69	17.0
sewage 75 cm^3^	7.5	339.0	56.00	32.80	0.00	10.02	28.8
sewage 100 cm^3^	7.5	452.6	56.00	35.00	0.00	7.82	44.5

MLVSS—2.910 g·dm^−3^, R—1.21, S—1.27.

**Table 7:** Influence of COD of industrial wastewater from polyvinyl acetate production on the nitrification inhibition in activated sludge under laboratory conditions.

Samples	pH	COD (mgO_2_·dm^−3^)	Concentration NH_4_-N (mg·dm^−3^)		Concentration NO_3_-N (mg·dm^−3^)		% IN
			Before Aeration	After 4 h of Aeration	Before Aeration	After 4 h of Aeration	
control	7.6	-	56.00	22.60	0.00	14.08	-
ATU	7.6	-	56.00	37.40	0.00	0.00	-
sewage 25 cm^3^	7.7	164.9	56.00	23.30	0.00	12.25	13.0
sewage 50 cm^3^	7.7	329.8	56.00	25.30	0.00	11.81	16.1
sewage 75 cm^3^	7.7	494.6	56.00	25.40	0.00	11.38	19.2
sewage 100 cm^3^	7.7	730.0	56.00	16.00	0.00	8.60	38.9

MLVSS—2.910 g·dm^−3^, R—1.21, S—1.27.

**Table 8:** Influence of COD of industrial wastewater from styrene production on the nitrification inhibition in activated sludge under laboratory conditions.

Samples	pH	COD (mgO_2_·dm^−3^)	Concentration NH_4_-N (mg·dm^−3^)		Concentration NO_3_-N (mg·dm^−3^)		% IN
			Before Aeration	After 4 h of Aeration	Before Aeration	After 4 h of Aeration	
control	7.6	-	56.00	21.30	0.00	12.96	-
ATU	7.6	-	56.00	48.90	0.00	0.73	-
sewage 25 cm^3^	7.4	60.5	56.00	29.70	0.00	8.51	37.5
sewage 50 cm^3^	7.4	121.1	56.00	30.16	0.00	8.32	39.1
sewage 75 cm^3^	7.4	181.6	56.00	30.48	0.00	7.96	42.1
sewage 100 cm^3^	7.4	242.1	56.00	32.19	0.00	7.61	45.1

MLVSS—5.880 g·dm^−3^, R—0.52, S—1.17.

**Table 9:** Influence of COD of industrial wastewater from solvent (butyl acetate, ethyl acetate) production on the nitrification inhibition in activated sludge under laboratory conditions.

Samples	pH	COD (mgO_2_·dm^−3^)	Concentration NH_4_-N (mg·dm^−3^)		Concentration NO_3_-N (mg·dm^−3^)		% IN
			Before Aeration	After 4 h of Aeration	Before Aeration	After 4 h of Aeration	
control	7.6	-	56.00	22.60	0.00	14.08	-
ATU	7.6	-	56.00	37.40	0.00	0.00	-
sewage 25 cm^3^	7.5	122.9	56.00	25.70	0.00	7.05	49.9
sewage 50 cm^3^	7.5	245.6	56.00	27.20	0.00	6.99	50.4
sewage 75 cm^3^	7.5	368.7	56.00	27.70	0.00	6.61	53.1
sewage 100 cm^3^	7.5	491.6	56.00	28.20	0.00	6.03	57.2

MLVSS—2.910 g·dm^−3^, R—1.21, S—1.27.

**Table 10:** Influence of COD of industrial wastewater from owipian^®^ production on the nitrification inhibition in activated sludge under laboratory conditions.

Samples	pH	COD (mgO_2_·dm^−3^)	Concentration NH_4_-N (mg·dm^−3^)		Concentration NO_3_-N (mg·dm^−3^)		% IN
			Before Aeration	After 4 h of Aeration	Before Aeration	After 4 h of Aeration	
control	7.6	-	56.00	21.30	0.00	12.96	-
ATU	7.6	-	56.00	48.90	0.00	0.73	-
sewage 25 cm^3^	7.2	155.3	56.00	25.46	0.00	6.92	49.4
sewage 50 cm^3^	7.2	316.1	56.00	26.97	0.00	6.37	53.9
sewage 75 cm^3^	7.2	466.0	56.00	28.24	0.00	6.03	56.7
sewage 100 cm^3^	7.2	621.3	56.00	31.72	0.00	5.96	57.2

MLVSS—5.880 g·dm^−3^, R—0.52, S—1.17.

**Table 11:** Summary of statistical parameters of the linear regression analysis assessing the effect of COD in industrial wastewater on the nitrification inhibition.

Production Line	Equation Parameter Value	R	R^2^	F	*p*-Value
acrylonitrile rubbers	a = 0.0838	0.945	0.893	16.634	0.055
	b = −2.256				
styrene–butadiene rubbers	a = 0.0670	0.955	0.912	20.800	0.044
	b = 14.300				
emulsifiers	a = 0.0906	0.961	0.924	24.144	0.039
	b = 0.537				
polyvinyl acetate	a = 0.0450	0.931	0.867	13.013	0.068
	b = 2.425				
styrene	a = 0.0430	0.991	0.983	112.510	0.009
	b = 34.500				
solvents (butyl acetate, ethyl acetate)	a = 0.0200	0.950	0.903	18.546	0.049
	b = 46.501				
owipian^®^	a = 0.0170	0.948	0.900	17.895	0.052
	b = 47.689				

a—the value of the directional coefficient of the regression equation, b—value of the intercept of the regression equation, R—value of the correlation coefficient between the analyzed variables, R^2^—value of the determination coefficient, F—the value of the Fisher–Snedecor F statistics, jointly verifying the significance of the relationship between the analyzed variables, the significance of the slope coefficient of the regression equation and the significance of the determination coefficient R^2^, *p*-value—the value of the calculated test probability for the F-test.

## Data Availability

Not applicable.
